# Synthesis, Anti-Proliferative Activity Evaluation and 3D-QSAR Study of Naphthoquinone Derivatives as Potential Anti-Colorectal Cancer Agents

**DOI:** 10.3390/molecules23010186

**Published:** 2018-01-17

**Authors:** Julio Acuña, Jhoan Piermattey, Daneiva Caro, Sven Bannwitz, Luis Barrios, Jairo López, Yanet Ocampo, Ricardo Vivas-Reyes, Fabio Aristizábal, Ricardo Gaitán, Klaus Müller, Luis Franco

**Affiliations:** 1 Biological Evaluation of Promising Substances Group, Faculty of Pharmaceutical Sciences, University of Cartagena, 130014 Cartagena, Colombia; julio.acuna.gutierrez@gmail.com (J.A.); dcarof@unicartagena.edu.co (D.C.); luisba1121@gmail.com (L.B.); yocampob@unicartagena.edu.co (Y.O.); 2 Natural Products Group, Faculty of Pharmaceutical Sciences, University of Cartagena, 130014 Cartagena, Colombia; jpiermatteyditta@gmail.com (J.P.); jairodejesuslopez@gmaill.com (J.L.); rgaitani@unicartagena.edu.co (R.G.); 3 Institute of Pharmaceutical and Medicinal Chemistry, PharmaCampus, Westphalian Wilhelms University, Corrensstraße 48, D-48149 Münster, Germany; bannwitz@uni-muenster.de (S.B.); kmuller@uni-muenster.de (K.M.); 4 Theoretical and Computational Chemistry Group, Faculty of Natural and Exact Sciences, University of Cartagena, 130015 Cartagena, Colombia; rvivasr@unicartagena.edu.co; 5 CipTec Group, Faculty of Engineering, Fundación Tecnológico Comfenalco de Cartagena, 130015 Cartagena, Colombia; 6 Cancer Pharmacogenetics Research Group, National University of Colombia, 111321 Bogotá, Colombia; faaristizabalg@unal.edu.co

**Keywords:** colorectal cancer, naphthoquinone derivatives, anti-proliferative activity, 3D-QSAR

## Abstract

Colorectal cancer (CRC) is a disease with high incidence and mortality, constituting the fourth most common cause of death from cancer worldwide. Naphthoquinones are attractive compounds due to their biological and structural properties. In this work, 36 naphthoquinone derivatives were synthesized and their activity evaluated against HT-29 cells. Overall, high to moderate anti-proliferative activity was observed in most members of the series, with 15 compounds classified as active (1.73 < IC_50_ < 18.11 μM). The naphtho[2,3-*b*]thiophene-4,9-dione analogs showed potent cytotoxicity, 8-hydroxy-2-(thiophen-2-ylcarbonyl)naphtho[2,3-*b*]thiophene-4,9-dione being the compound with the highest potency and selectivity. Our results suggest that the toxicity is improved in molecules with tricyclic naphtho[2,3-*b*]furan-4,9-dione and naphtho[2,3-*b*]thiophene-4,9-dione systems 2-substituted with an electron-withdrawing group. A 3D-QSAR study of comparative molecular field analysis (CoMFA) was carried out, resulting in the generation of a reliable model (r^2^ = 0.99 and q^2^ = 0.625). This model allowed proposing five new compounds with two-fold higher theoretical anti-proliferative activity, which would be worthwhile to synthesize and evaluate. Further investigations will be needed to determine the mechanism involved in the effect of most active compounds which are potential candidates for new anticancer agents.

## 1. Introduction

According to the World Health Organization (WHO), in 2012, 14 million cases and 8.2 million deaths were attributed to cancer, a disease characterized by abnormal and uncontrolled growth of cells (tumor) and colonization of tissues (metastasis); which is documented as the second cause of mortality after cardiovascular diseases, and is expected to surpass 20 million new cases by 2025 [1]. The colorectal cancer (CRC) is the third most commonly diagnosed cancer worldwide, after lung and breast cancer, and constitutes the fourth cause of cancer death [2]. CRC, as other types of cancer, is the result of accumulation of genetic and epigenetic mutations in specific oncogenes and/or tumor suppressor genes causing the formation of adenocarcinomas, with about 10–30% of patients having a family history of the disease (familial CRC) and only 5–6% caused by a defined hereditary syndrome, whereas about 75% of patients with CRC have sporadic adenomatous colorectal polyps and 3.7% extensive ulcerative colitis [3,4]. The molecular understanding of CRC carcinogenesis continues to rapidly advance, allowing the improvement of prediction, early diagnostic, prognosis, and clinical management [5]. Current treatment of CRC employs surgical resection combined with chemotherapy, cytotoxic drugs, and radiotherapy [6]. These therapies are often ineffective, mainly due to the resistance developed by cancer stem cells, leading to recurrence in a significant amount of patients with late-stage CRC, as well as a high incidence of serious and even life-threatening adverse effects that significantly reduce the quality of life of patients [7,8]. Given the serious adverse effects, the elevated costs associated with the treatment, and the low efficiency of current chemotherapy; there is a clear necessity to discover new anticancer agents that are more effective, safe and affordable.

Naphthoquinones are molecules widely distributed in nature that possess a broad spectrum of biological activities, including anticancer activity, which has stimulated their study and recognition as privileged structures in medicinal chemistry [9,10]. Cytotoxic activity exhibited by quinone derivatives is mediated mainly by several mechanisms, including the inhibition of DNA topoisomerase-II, an enzyme essential in the replication of DNA and the condensation and segregation of chromosomes; as well as through the formation of semiquinones and superoxide radicals, which contribute to the production of hydroxyl radical, the main source of oxidative generated damage to cellular DNA [11,12,13]. Interestingly, medicinal plants containing naphthoquinones, such as *Tabebuia* spp. (locally known as lapacho, pau-d’arco, roble or guayacan), are extensively employed in South America for their analgesic, anti-inflammatory, and anti-neoplastic properties. Furthermore, many clinically important antitumor agents, such as anthracyclines (e.g., daunorubicin and doxorubicin), mitomycin, mitoxantrone and saintopin, contain the quinone nucleus. Nevertheless, little information is available regarding their ability to treat CRC. In addition, studies aiming to design or optimize the structure of naphthoquinones to improve specifically their bioactivity or selectivity against CRC are infrequent.

In this work, we synthetized a series of 36 naphthoquinone derivatives, analogous of quinones isolated from *Tabebuia* spp., which were designed to include a wide arrange of chemical modifications (alkylation, amination, epoxidation, halogenation, and cycloaddition) to evaluate the changes in their cytotoxicity against CRC cells. Furthermore, a three-dimensional quantitative structure–activity relationship (3D-QSAR) was carried out to correlate the bioactivity with the chemical structure.

## 2. Results and Discussion

### 2.1. Chemistry

**Figure 1:**
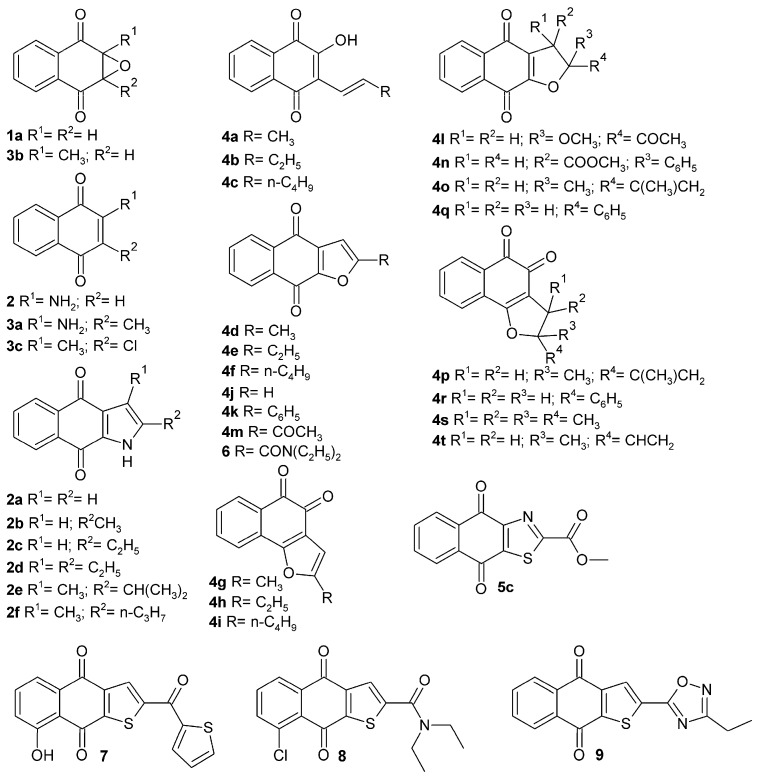
Naphthoquinone derivatives designed, synthesized and evaluated in this study.

**Scheme 1:**
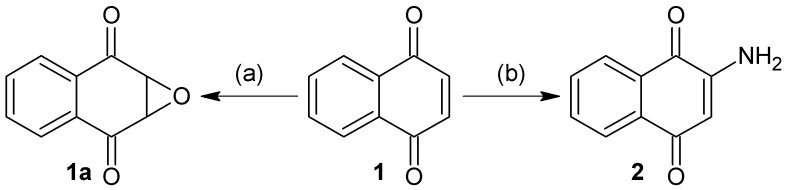
Synthesis of (**1a** and **2**). Reagents and conditions: (**a**) EtOH, H_2_O_2_, Na_2_CO_3_; and (**b**) EtOH, NaN_3_, H_2_O, HCl, 50 °C, 16 h.

**Scheme 2:**
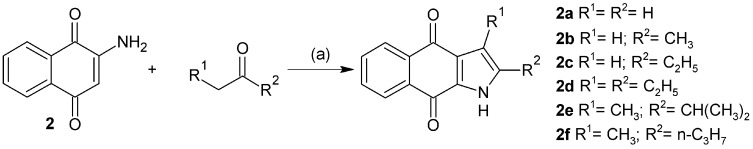
Synthesis of (**2a**–**2f**). Reagents and conditions: (**a**) Mn(AcO)_3_, CH_3_CN, 80 °C, 39 h.

**Scheme 3:**
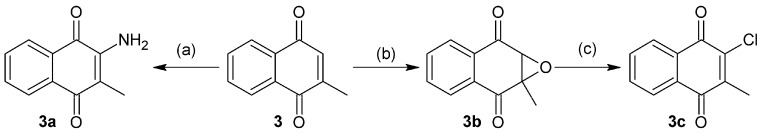
Synthesis of (**3a**–**3c**). Reagents and conditions: (**a**) EtOH, NaN_3_, H_2_O, HCl, 50 °C, 16 h; (**b**) EtOH, H_2_O_2_, Na_2_CO_3_; and (**c**) HCl, MeOH, 70 °C, 3 h.

**Scheme 4:**
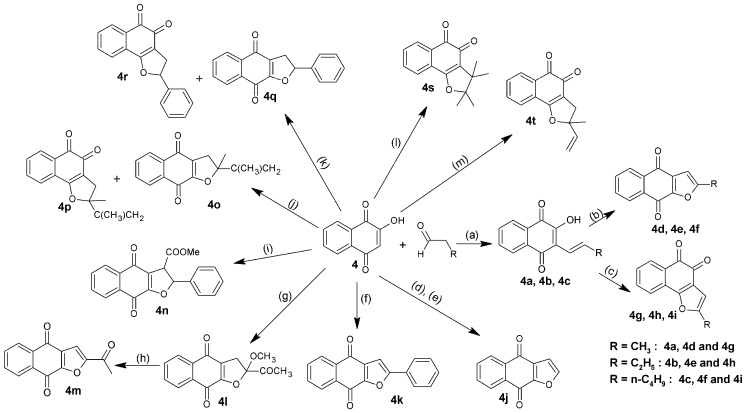
Synthesis of (**4a**–**4t**). Reagents and conditions: (**a**) AcOH, HCl, 60 °C; (**b**) Hg(AcO)_2_, AcOH, 65 °C; EtOH, HClconc., 65 °C, 2 h; (**c**) Hg(AcO)_2_, AcOH, 65 °C; EtOH, HCldil., 65 °C, 15 min; (**d**) vinyl acetate, acetone, uv, 7 h; (**e**) p-TsOH, dry benzene, reflux; (**f**) phenylacetylene, CH_3_CN, CAN, 0 °C, 20 min; (**g**) 2,3-dimethoxy-1,3-butadiene, CH_3_CN, CAN, 0 °C, 6 h; (**h**) benzene, DBU, r.t., 5 h; (**i**) acetone, methyl (2*E*) 2-phenylpropionate, uv, 22 h; (**j**) 2,3-dimethoxy-1,3-butadiene, CH_3_CN, CAN, Na_2_CO_3_, 0 °C, 3 h; (**k**) styrene, CH_3_CN, CAN, 0 °C, 20 min; (**l**) 2,3-dimethyl-2-butene, CH_3_CN, CAN 0 °C, 20 min; and (**m**) isoprene, CH_3_CN, CAN, 0 °C, 3 h.

**Scheme 5:**
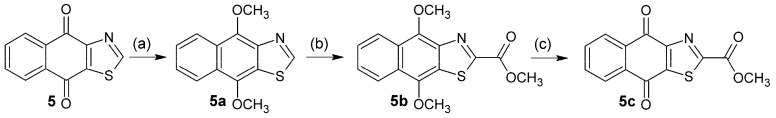
Synthesis of (**5a**–**5c**). Reagents and conditions: (**a**) Na_2_S_2_O_4_, Bu_4_NBr, THF, KOH, Me_2_SO4, N2; (**b**) C_2_CO_3_, CO_2_, DMF, 125 °C, 400 mbar, 16 h; CH_3_I, 60 °C, 2 h; and (**c**) (NH_4_)_2_[Ce(NO_3_)_6_], MeCN, H_2_O, 0 °C.

The compounds evaluated in this work (Figure 1) were synthesized using several starting materials, as outlined in Scheme 1, Scheme 2, Scheme 3, Scheme 4 and Scheme 5. Naphthalene-1,4-dione (**1**) was subjected to epoxidation with hydrogen peroxide to yield compound **1a**. On the other hand, **1** was subjected to an amination reaction by means of Michael-type 1,4-conjugate addition to give **2** (Scheme 1). Subsequently, an oxidative free radical reaction was performed with either aldehydes or simple ketones in the presence of manganese (III) acetate to afford a series of indolenaphthoquinones (**2a**–**2f**) (Scheme 2) [14]. Compound **3a** was obtained using 2-methylnaphthalene-1,4-dione (**3**) as starting material and a similar procedure as that employed to synthetize **2**. Epoxidation reaction of **3** afforded **3b**, followed by an acid hydrolysis to yield **3c** (Scheme 3).

Condensation of 2-hydroxynaphthalene-1,4-dione (**4**) with aldehydes in acidic condition was carried out as described by Hooker [15] to afford compounds **4a**, **4b**, and **4c**. These compounds were oxidized and cyclized by mercuric acetate in harsh reaction conditions, concentrated hydrochloric acid, 65 °C and 2 h of reaction time, to afford **4d**, **4e**, and **4f**, respectively. Alternatively, milder reaction conditions, i.e. diluted hydrochloric acid, 65 °C and 15 min of reaction time, were used to obtain **4g**, **4h**, and **4i** [16]. Cycloaddition reactions of **4** with different reagents were carried out to obtain the compounds **4j**–**4t**. The compound **4m** was obtained by the hydrolysis of **4l** (Scheme 4). For the synthesis of naphthothiazole compounds, naphtho[2,3-*d*]thiazole-4,9-dione (**5**) [17] was subjected to reductive methylation to afford **5a**. This was directly carboxylated and converted to the ester in a one-pot procedure with carbon dioxide and methyl iodide in the presence of cesium carbonate [18] to give methyl ester **5b**. Final oxidation and ether cleavage using diammonium cerium(IV) nitrate provided the target structure **5c** (Scheme 5). Finally, following previously reported procedures, the compound *N*,*N*-diethyl-4,9-dioxo-4,9-dihydronaphtho[2,3-*b*]furan-2-carboxamide (**6**) [19], as well as the naphthothiophene derivatives 8-hydroxy-2-(thiophen-2-ylcarbonyl)naphtho[2,3-*b*]thiophene-4,9-dione (**7**) [20], 8-chloro-*N*,*N*-diethyl-4,9-dioxo-4,9-dihydronaphtho[2,3-*b*]thiophene-2-carboxamide (**8**) [21], and 2-(3-ethyl-1,2,4-oxadiazol-5-yl)naphtho[2,3-*b*]thiophene-4,9-dione (**9**) [22] were synthesized.

### 2.2. Biological Evaluation

These as-synthesized quinone derivatives were further utilized to investigate their cytotoxic effect against a human colorectal cancer cell line (HT-29) and normal dermal fibroblasts (PCS-201-012). The toxicity of the thirty-six compounds was determined in vitro by means of the MTT assay. Doxorubicin, a drug widely used to treat different types of cancer, was employed as a positive control, whereas the solvent (DMSO) was used as negative control. Results of the anti-proliferative activity of quinone derivatives against HT-29 cells, expressed as the IC_50_ values, as well as the selectivity index, are presented in Table 1. Out of the 36 compounds tested, 25 could reduce the growth of the cells to 50% or less, at concentrations equal to or lower than 100 μM, and thus classified according to their cytotoxic activity into three categories: active (IC_50_ ≤ 20 µM), moderately active (20 µM < IC_50_ ≤ 100 µM) and inactive (IC_50_ > 100 µM). In general, moderate to high cytotoxic activity was observed for most of the members of the series. Results showed 15 compounds classified as active (**1a**, **3c**, **4d**, **4g**, **4h**, **4i**, **4j**, **4m**, **4r**, **4s**, **5c**, **6**, **7**, **8**, and **9**) with IC_50_ values ranging from 1.73 to 18.11 μM, and 10 compounds classified as moderately active (**2**, **2c**, **3b**, **4e**, **4f**, **4l**, **4o**, **4p**, **4q**, and **4t**) with IC_50_ values ranging from 23.73 to 88.1 μM, while the remaining compounds were inactive.

The naphtho[2,3-*b*]thiophene-4,9-dione analogs (**7**, **8**, and **9**) showed a potent activity against HT-29 cells. Notably, compound **7** showed the strongest activity against this cell line with an IC_50_ of 1.73 μM, and the highest selectivity index. On the other hand, it is important to highlight the activity shown by the furanonaphthoquinone derivatives (**4a**–**4t** and **6**), which were classified within the moderately (**4e** and **4f**) and highly active (**4d**, **4g**, **4h**, **4i**, **4j**, **4m**, and **6**) groups. In contrast, the groups of 1,4-naphthoquinone, dihydrofuran-naphthoquinone and benzo[*f*]indole-4,9-dione derivatives had proportionally fewer molecules with cytotoxic potential compared to the above-mentioned analogs. In the case of the naphthoquinone group, only compounds **1a** and **3c** were classified as active, suggesting that carbon chain substituents in the B ring decrease the activity of these derivatives (**2** vs. **3a** and **1a** vs. **3b**). Dihydrofuran-naphthoquinone compounds showed mainly moderately activity against HT-29 cells, with only compounds **4r** and **4s** classified as active molecules. Alternatively, the benzo[*f*]indole-4,9-dione derivatives showed the lowest cytotoxic potential of the series, without molecules categorized as active and only the **2c** derivative classified as moderately active. Thus, the presence of an indole moiety in naphthoquinones, within a tricyclic system, had a negative effect on their cytotoxic activity against HT-29 colorectal cancer cell line.

Regarding the selectivity index of the synthesized series, only three derivatives showed selectivity towards CRC cells (**2c**, **4o**, and **7**), demonstrating that HT-29 cells are resistant to naphthoquinone derivatives, but specific modifications might improve their bioactivity. Furthermore, it was evident that cycloaddition to the basic naphthoquinone moiety potentiates the selectivity, which might be related to enhanced stability or uptake in the HT-29 cells when compared to normal fibroblasts.

In general, our results showed a clear structure–activity relationship (SAR). It is observed that the presence of a furan or thiophene ring on the naphthoquinone scaffold increases the anti-proliferative activity against CRC cells. Probably, these structural changes might lead to enhanced: (1) compound stability in the presence of serum; (2) uptake into HT-29 cells; or (3) affinity for an unknown target. Additionally, electron-attracting groups in 2-position of the furan or thiophene ring improve the bioactivity, as observed in the quinone derivatives (**4m**, **5c**, **6**, **7**, **8**, and **9**) which showed IC_50_ values between 1.73 and 13.9 μM. On the contrary, furanonaphthoquinones 2-substituted with an electron releasing group (alkyl substituents) showed reduced activity (**4j** vs. **4d**, **4e**, and **4f**). This structure–activity relationship was also observed by Wang et al. [23], who evaluated several quinone derivatives against a panel of four cancer cell lines, including the furanonaphthoquinone **4m**, observing drastic changes on the activity in function of the substituent present in the mentioned position. Similarly, Inagaki et al. [24], evaluated the cytotoxic activity of furanonaphthoquinone analogs against two cancer cell lines, observing stronger cytotoxicity in compounds with a 2-acetyl substituted furan ring. Likewise, a series of analogs substituted at the 2-position of the tricyclic naphtho-[2,3-*b*]furan-4,9-dione system, including the compounds **4d**, **4e**, **4j** and **6**, were evaluated to determine the ability to suppress keratinocyte hyperproliferation, showing the importance of 2-electron withdrawing substituents [19]. Furthermore, it was observed that quinone derivatives with a tricyclic system in an angular conformation (4,5-diones) showed higher activity than those with a linear conformation (4,9-diones), suggesting that the spatial configuration is important for the cytotoxic activity; which agrees with the report of Kongkathip et al. [25].

### 2.3. 3D-QSAR

**Figure 2:**
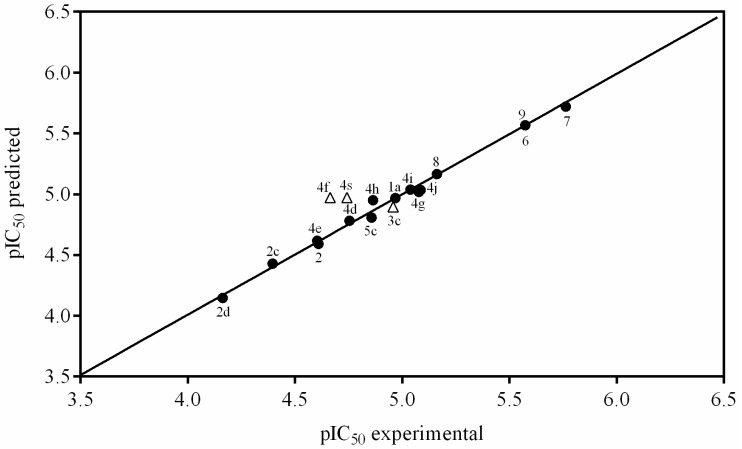
Correlation between predicted and experimental cytotoxic activity (pIC_50_) of training (dark circles) and test (light triangles) sets using the best comparative molecular field analysis (CoMFA) model.

To better understand the structure activity relationship, a three-dimensional quantitative structure–activity relationship (3D-QSAR) study of comparative molecular field analysis (CoMFA), using the module QSAR Project Manager of SYBYL-X 2.0 (Tripos, St. Louis, MO, USA), was carried out using 18 quinone derivatives with defined stereochemistry, including all ranges of activity. The cytotoxic activity of molecules, expressed as −logIC_50_ (pIC_50_), was related to the independent variables using the partial least squares (PLS) analysis. The statistical parameters of CoMFA model indicated good correlation and predictive power (r^2^ = 0.99, q^2^ = 0.625; for four components); the contribution of steric and electrostatic fields was found to be 40:60. Experimental and predicted values of pIC_50_ obtained from the best CoMFA model for each compound of the compounds included in the study are presented in Table 2. In addition, the correlation plot between experimental versus predicted cytotoxic activity for the training set and the test set is shown in Figure 2. Our results demonstrate a good predictability for all molecules with the absence of outliers. In addition, all parameters of the CoMFA model show some reliability and feasibility to ensure the design of new quinone derivatives with potent cytotoxicity against CRC cells.

**Figure 3:**
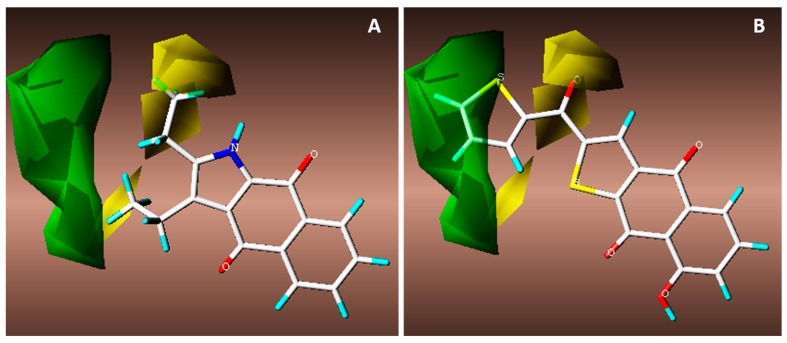
Steric contour maps generated by comparative molecular field analysis (CoMFA) for: (**A**) 2,3-diethyl-1*H*-benzo[*f*]indole-4,9-dione (**2d**), the least active compound of the series; and (**B**) hydroxy-2-(thiophen-2-ylcarbonyl)naphtho[2,3-*b*]thiophene-4,9-dione (**7**), the most active.

**Figure 4:**
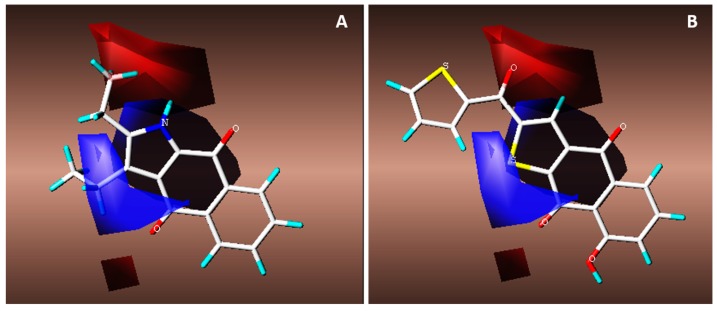
Electrostatic contour maps generated by comparative molecular field analysis (CoMFA) for: (**A**) 2,3-diethyl-1*H*-benzo[*f*]indole-4,9-dione (**2d**), the least active compound of the series; and (**B**) hydroxy-2-(thiophen-2-ylcarbonyl)naphtho[2,3-*b*]thiophene-4,9-dione (**7**), the most active.

CoMFA is a shape-dependent 3D-QSAR technique which is sensitive to both conformational and configurational changes within a series of molecules [26]. In CoMFA method, results are presented as contour maps that correlate the change in biological activity with the molecular field value. In this work, compounds **2d** and **7** were selected to explain the field contributions of different properties obtained from the CoMFA analysis, since they have the extreme values of cytotoxic activity. The steric and electrostatic contour CoMFA fields are shown in Figure 3 and Figure 4, respectively, to illustrate the most influential areas for biological activity. In the CoMFA steric field, the green contours represent the region favorable regions where bulky groups increase the cytotoxicity, whereas unfavorable regions are represented by yellow contours (Figure 3). In the case of the electrostatic CoMFA fields, red contours indicate regions where electronegative groups enhance the bioactivity, while blue contours show the regions where electropositive groups improve the activity (Figure 4).

As shown in Figure 3, CoMFA steric map shows the presence of a thiophenyl group (D ring) of compound **7** in the favorable zone to bulky group (green polyhedron in Figure 3B). In contrast, compound **2d** has no presence of substituent in this area, while an ethyl substituent is oriented towards the yellow polyhedron, that constitute unfavorable zones for bulky substituents (Figure 3A), negatively affecting its anti-proliferative activity against this cell line. Alternatively, CoMFA electrostatic map shows that while molecule **7** does not have any electronegative atom within the red favorable contour, it possesses a carbonyl group in proximity to the red polyhedron, which in turn improves its bioactivity. Notably, this substituent is absent in compound **2d**. Furthermore, the presence of a nitrogen atom in the ring C of compound **2**, in proximity to a blue contour, explains the reduction of its anti-proliferative activity in comparison to compound **7**, which has sulfur, a less electronegative atom.

#### Design of New Quinone Derivatives

**Figure 5:**
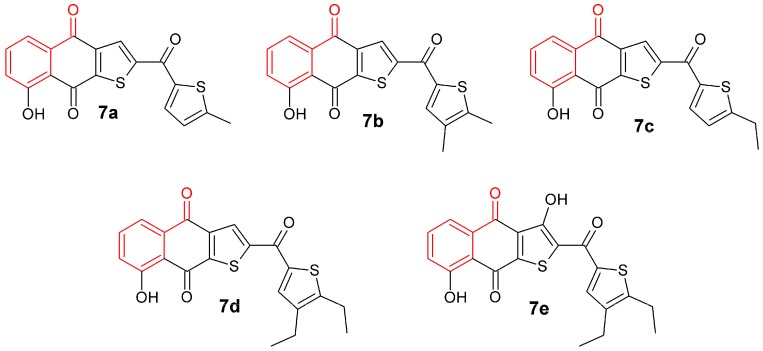
Chemical structure of the molecules designed according to comparative molecular field analysis (CoMFA) analysis.

Analytical interpretation of the 3D-QSAR models resulted in the identification of key structural features which could be exploited for improving the potency of the molecule **7**, used as a reference for being the most active of the series. Therefore, a library of new structures based on compound **7** was designed, where methyl or ethyl substituents were added to the thiophenyl group (D ring) attached to the naphtho[2,3-*b*]thiophene-4,9-dione structure, to increase the bulkiness in this zone (green polyhedron). Thus, five new compounds are proposed: 2-[(5-methylthiophen-2-yl)carbonyl]naphtha[2,3-*b*]thiophene-4,9-dione (**7a**), 2-[(4,5-dimethylthiophen-2-yl)carbonyl]naphtho[2,3-*b*]thiophene-4,9-dione (**7b**), 2-[(5-ethylthiophen-2-yl)carbonyl]naphtho[2,3-*b*]thiophene-4,9-dione (**7c**), 2-[(4,5-diethylthiophen-2-yl)carbonyl]naphtho[2,3-*b*]thiophene-4,9-dione (7d), and 2-[(4,5-diethylthiophen-2-yl)carbonyl]-3-hydroxynaphtho[2,3-*b*]thiophene-4,9-dione (**7e**) (Figure 5). These molecules were digitized, optimized and aligned using the same methodology described for the experimental molecules, and a two-fold improved theoretical anti-proliferative activity against HT-29 cells was calculated in comparison to the parental compound (**7**) (Table 3).

The first modification to **7** was the introduction of a methyl group in the C3 of the thiophenyl ring resulting in molecule **7a**, which showed an increased activity. Nevertheless, when a double methyl substitution was made in C3 and C4, the new molecule **7b** presented a theoretical activity lower than the analog with only one substitution (**7a**), although higher than the parental compound (**7**). The inclusion of ethyl groups in these positions resulted in better activity, giving compounds **7c** and **7d**, with one and two substitutions, respectively. Interestingly, when the ethyl group was employed, the di-substituted compound was the most active. Finally, molecule **7d** was modified with the insertion of a hydroxyl (OH) group in the favorable zone for electronegative groups (red polyhedron), specifically at 3-position of the thiophene (ring C), to obtain the compound **7e**. Unexpectedly, this modification produced a decrease of the bioactivity in comparison with molecule **7d**.

To sum up, a library of five new molecules with higher predicted cytotoxicity was proposed. All the designed molecules presented a theoretical IC_50_ value lower than 1 μM, demonstrating the efficacy of the 3D-QSAR model for the design of new and more potent cytotoxic naphthoquinones against CRC cell line HT-29.

## 3. Materials and Methods

### 3.1. General Information

All the reagents were ACS grade and purchased from Sigma-Aldrich (St. Louis, MO, USA); the solvents were obtained from J.T. Baker (Center Valley, PA, USA), and the Silica 60-230, analytical and preparative thin layer (TLC) chromatography plates from Merck (Merck Millipore, Billerica, MA, USA). Melting points were measured with a Fisher/Johns melting point apparatus (Thermo Fisher Scientific, Waltham, MA, USA) and are uncorrected. The IR spectra were determined on a FTIR-8400S spectrometer (Shimadzu Corporation, Kyoto, Japan) using KBr disks. The NMR spectra were acquired on Bruker spectrometers (300 MHz, 400 MHz or 600MHz) (Bruker, Billerica, MA, USA and Bruker, Rheinstetten, Germany) using deuterated chloroform as solvent, and trimethylsilane (TMS) as internal standard. The mass spectra were obtained from an Agilent HP 6890 gas chromatograph equipped with an HP 5973 selective mass detector (Agilent Technologies, Santa Clara, CA, USA). TLC, MS, NMR, and/or HPLC analytical data confirmed that purity of test compounds was ≥95%.

### 3.2. Synthesis of Naphthoquinone Derivatives

#### 3.2.1. Synthesis of 1*a*,7*a*-Dihydronaphtho[2,3-*b*]oxirene-2,7-dione (**1a**)

To a solution of 200 mg of **1** in 10 mL of hot ethanol was added 3 mL of H_2_O_2_ (30%) with 0.2 g of anhydrous sodium carbonate; the reaction mixture was allowed to cool until the yellow color disappeared completely. Then, 50 mL of cold water was added and the mixture was immersed in an ice bath to form a white precipitate, which was filtered in vacuum to afford white needles; 22.63% yield; m.p. 125 °C; FTIR (KBr) ν: 3000, 2968, 1680, 1590, 1260 cm^−1^; ^1^H-NMR (600 MHz, CDCl_3_): δ 8.20 (m, 2H), 7.80 (m, 2H), 3.90 (s, 2H) ppm; ^13^C-NMR (600 MHz, CDCl_3_): δ 191.150 (C-1; C-4), 135.162 (C-9; C-10), 134.72 (C-6; C-7); 127.673 (C-5; C-8), 55.74 ppm (C-2; C-3); MS *m*/*z* 174 [M^+^, C_10_H_6_O_3_].

#### 3.2.2. Synthesis of 2-Aminonaphthalene-1,4-dione (**2**)

To a previously stirred solution of 2 g of **1** in 100 mL of ethanol was added 4.74 g of sodium azide in 34 mL of distilled water, then adjusted to pH 4 with HCl and allowed to stir for 15 h at reflux temperature. After completion of the reaction time, extraction was performed with ethyl acetate (3 × 100 mL). The organic extracts were washed with saline solution, dried over anhydrous Na_2_SO_4_ and concentrated. The residue was crystallized from a mixture of diethyl ether/hexane to afford an orange powder; 90% yield; m.p. 207.5 °C; FTIR (KBr) ν: 3381, 3191, 3065, 2923, 1685, 1613 cm^−1^; ^1^H-NMR (300 MHz, CDCl_3_): δ 8.10 (m, 2H), 7.67 (m, 2H), 6.0 (s, 1H), 5.12 (s, 2H) ppm; ^13^C-NMR (300 MHz, CDCl_3_): δ 186.20 (C-4), 184.01 (C-1), 151.52 (C-2), 136.34 (C-9), 135.31 (C-10), 133.80 (C-6), 132.52 (C-7), 126.90 (C-8), 126.71 (C-5), 104.90 (C-3) ppm; MS *m*/*z* 173 [M^+^, C_10_H_7_O_2_N].

#### 3.2.3. General Synthesis of Benzo[*f*]indole-4,9-diones (**2a**–**2f**)

A mixture of 109 mg (0.64 mmol) of **2**, 6.4 mmol of ketone (or aldehyde) and 1.04 g (3.88 mmol) of manganese (III) acetate in 10 mL of acetonitrile was heated at 80 °C for 16 h; at this point, 1.04 g (3.88 mmol) of manganese (III) acetate was added again and heated for another 23 h, then the mixture reaction was diluted with 100 mL of ethyl acetate, washed with 50 mL of saturated aqueous sodium bisulfate, three time with 50 mL of water, and dried over Na_2_SO_4_. The solvent was evaporated under reduce pressure and the crude product was purified by column chromatography (SiO_2_; CH_2_Cl_2_/hexane, 1/1), followed by crystallization (ethyl acetate–hexane).

*1H-Benzo[f]indole-4,9-dione* (**2a**). Yellow powder; 44% yield; m.p. 281–283 °C; FTIR (KBr) ν: 3424, 3292, 1655, 1387 cm^−1^; ^1^H-NMR (300 MHz, DMSO-*d*): δ 8.01 (m, 2H), 7.75 (m, 2H), 6.42 (s, 1H), 2.3 (s, 3H) ppm; ^13^C-NMR (300 MHz, DMSO-*d*): δ 180.76 (C-4), 175.16 (C-9), 134.08 (C-12), 133.95 (C-10), 133.86 (C-11), 133.55 (C-7), 132.73 (C-6), 128.39 (C-2), 127.53 (C-8), 126.73 (C-5), 126.42 (C-13), 108.55 (C-3) ppm; MS *m/z* 197.19 [M+, C_12_H_7_O_2_N].

2-Methyl-1*H*-benzo[*f*]indole-4,9-dione (**2b**). Yellow powder; 34% yield; m.p. 302–304 °C; FTIR (KBr) ν: 3454, 3218,1648, 1223 cm^−1^; ^1^H-NMR (300 MHz, DMSO-*d*): δ 8.03 (m, 2H), 7.7 (m, 2H), 7.35 (s, 1H), 6.69 (s, 1H) ppm; ^13^C-NMR (300 MHz, DMSO-*d*): δ 180.80 (C-4), 174.24 (C-9), 139.45 (C-12), 133.92 (C-10), 133.79 (C-11), 133.68 (C-7), 133.64 (C-6), 131.89 (C-2), 128.17 (C-5), 126.57, 126.26 (C-8), 126.0 (C-13), 107.04 (C-3), 13.13 (C-14) ppm; MS *m/z* 211.21 [M^+^, C_13_H_9_O_2_N].

2-Ethyl-1*H*-benzo[f]indole-4,9-dione (**2c**). Yellow powder; 53% yield; m.p. 200–207 °C; FTIR (KBr) ν: 3485, 3201, 1652, 1393 cm^−1^; ^1^H NMR (300 MHz, DMSO- *d*) δ 8.01 (m, 2H), 7.74 (m, 2H), 7.15 (s, 1H), 2.76 (q, *J* = 7.4 Hz, 2H), 1.19 (t, *J* = 7.4 Hz, 3H) ppm; ^13^C-NMR (300 MHz, DMSO-*d*): δ 181.47 (C-4), 175.06 (C-9), 134.59 (C-12), 133.72 (C-10), 133.65 (C-11), 133.52 (C-7), 133.47 (C-6), 129.08 (C-2), 126.95 (C-5), 126.49 (C-8), 126.16 (C-13), 124.13 (C-3), 19.28 (C-14), 14.79 (C-15); MS *m*/*z* 225.24 [M^+^, C_14_H_11_O_2_N].

2,3-Diethyl-1*H*-benzo[f]indole-4,9-dione (**2d**). Orange powder; 36% yield; m.p. 205–209 °C; FTIR (KBr) ν: 3446, 3214, 1655, 1467 cm^−1^; ^1^H NMR (300 MHz, DMSO-*d*) δ 13.17 (s, 1H), 8.06 (d, *J* = 4.5 Hz, 2H), 7.81 (m, 2H), 3.03 (q, *J* = 7.1 Hz, 2H), 2.70 (q, *J* = 7.4 Hz, 2H), 1.1 (m, 6H) ppm; ^13^C-NMR (300 MHz, DMSO-*d*): δ 180.91 (C-4), 175.36 (C-9), 146.74 (C-12), 134.80 (C-10), 134.25 (C-11), 133.99 (C-7), 132.69 (C-6), 131.73 (C-2), 127.10 (C-5), 126.19 (C-8), 124.32 (C-13), 121.34 (C-3), 19.91 (C-14), 19.02 (C-16), 14.74 (C-15), 8.96 (C-17); MS *m*/*z* 253.29 [M^+^, C_16_H_15_O_2_N].

3-Methyl-2-(propan-2-yl)-1*H*-benzo[f]indole-4,9-dione (**2e**). Orange powder; 42% yield; m.p. 245–248 °C; FTIR (KBr) ν: 3423, 3198, 1655, 1378 cm^−1^; ^1^H NMR (300 MHz, DMSO-*d*) δ 7.99 (m, 2H), 7.74 (m, 2H), 2.65 (m, 1H), 2.22 (s, 3H), 1.01 (m, 6H) ppm; ^13^C-NMR (300 MHz, DMSO-*d*): δ 181.62 (C-4), 173.85 (C-9), 136.24 (C-12), 134.29 (C-10), 133.61 (C-11), 133.52 (C-7), 130.74 (C-6), 126.42 (C-2), 126.01 (C-5), 125.22 (C-8), 124.64 (C-13), 106.24 (C-3), 17.75 (C-14), 15.18 (C-15; C-16), 10.82 (C-17); MS *m*/*z* 253.31 [M^+^, C_16_H_15_O_2_N].

3-Methyl-2-propyl-1*H*-benzo[f]indole-4,9-dione (**2f**). Orange powder; 41% yield; m.p. 266–270 °C; FTIR (KBr) ν: 3424, 1655 cm^−1^; ^1^H NMR (300 MHz, DMSO-*d*) δ 7.99 (m, 2H), 7.72 (m, 2H), 2.60 (m, 2H), 2.26 (s, 3H), 1.64 (m, 2H), 1.15 (t, 3H) ppm; ^13^C-NMR (300 MHz, DMSO-*d*): δ 181.91 (C-4), 173.95 (C-9), 143.12 (C-12), 134.43 (C-10), 133.87 (C-11), 133.47 (C-7), 133.38 (C-6), 131.25 (C-2), 126.34 (C-5), 126.03 (C-8), 125.34 (C-13), 117.84 (C-3), 25.54 (C-14), 18.80 (C-15), 14.32 (C-17), 10.07 (C-16); MS *m*/*z* 253.29 [M^+^, C_16_H_15_O_2_N].

#### 3.2.4. Synthesis of 2-Amino-3-methylnaphthalene-1,4-dione (**3a**)

Compound **3a** was obtained with the same procedure for the synthesis of **2**, using 2-methyl-1,4-naphthoquinone as starting material and 36 h for reaction time. This afforded a red powder; 68.66% yield; m.p. 158 °C; FTIR (KBr) ν: 3455, 3411, 2940, 1670, 1628, 1590 cm^−1^; ^1^H-NMR (600 MHz, CDCl_3_): δ 8.1 (m, 2H), 7.7 (m, 2H), 5.15 (s, 2H), 2.01 (s, 3H) ppm; ^13^C-NMR (600 MHz, CDCl_3_): δ 182.95 (C-1), 181.22 (C-4), 145.32 (C-2), 134.56 (C-10), 133.22 (C-9), 132.06 (C-7), 130.48 (C-6), 126.27 (C-8), 125.82 (C-5), 113.29 (C-3), 12.981 (C-11) ppm; MS *m*/*z* 187.19 [M^+^, C_11_H_9_O_2_N].

#### 3.2.5. Synthesis of 1*a*-Methyl-1*a*,7*a*-dihydronaphtho[2,3-*b*]oxirene-2,7-dione (**3b**)

To a solution of 1.25 g of **3** in 20 mL of hot ethanol was added 7.5 mL of H_2_O_2_ (30%) with 0.5 g of sodium carbonate. The reaction mixture was allowed to reach room temperature (rt) until the yellow color of the quinone completely disappeared. Then, 125 mL of cold water was added and the mixture was immersed in an ice bath to form a white precipitate which was filtered in vacuum to afford white solid; 65.5% yield; m.p. 138 °C; FTIR (KBr) ν: 3095, 3039, 2995, 2939, 1680, 1590, 1260 cm^−1^; ^1^H-NMR (600 MHz, CDCl_3_): δ 8.04 (m, 2H), 7.76 (m, 2H), 3.86 (s, 1H), 1.75 (s, 1H) ppm; ^13^C-NMR (600 MHz, CDCl_3_): δ 192.36 (C-1), 192.19 (C-4), 134.99 (C-9), 134.80 (C-10), 132.56 (C-7), 132.44 (C-6), 127.89 (C-8), 127.25 (C-5), 61.82 (C-2), 59.67 (C-3), 15.13 (C-11) ppm; MS *m/z* 188.18 [M^+^, C_11_H_8_O_3_].

#### 3.2.6. Synthesis of 2-Chloro-3-methylnaphthalene-1,4-dione (**3c**)

To 100 mL of HCl (5%) in methanol was added 280 mg of **3b** and refluxed at 70 °C for 3h. The obtained mixture was extracted with ethyl acetate (3 × 100 mL), then the organic phase was filtered in the presence of anhydrous Na_2_SO_4_, the solvent was removed by rotaevaporation. The obtained residue was purified by column chromatography (SiO_2_; CH_2_Cl_2_) to afford yellow needles; 25.5% yield; m.p. 158 °C; FTIR (KBr) ν: 3095, 3038, 3000, 2957, 1669, 1587, 705 cm^−1^; ^1^H-NMR (600 MHz, CDCl_3_): δ 8.10 (m, 2H), 7.71 (m, 2H), 2.28 (s, 3H) ppm; ^13^C-NMR (600 MHz, CDCl_3_): δ 183.05 (C-1), 178.01 (C-4), 145.32 (C-3), 143.77 (C-2), 134.59 (C-6), 134.35 (C-7), 132.14 (C-10), 131.83 (C-9), 127.62 (C-5), 127.44 (C-8), 14.92 (C-11) ppm; MS *m*/*z* 206.63 [M^+^, C_11_H_7_O_2_Cl].

#### 3.2.7. General Synthesis of Hydroxynaphthalene-1,4-diones (**4a**–**4c**)

Two grams of **4** were dissolved in 30 mL of glacial acetic acid, and then 2 mL of hydrochloric acid and 7 mL of aldehyde (propanal, butanal or hexanal) were added. The mixture was heated at 60 °C until disappearance of the starting compounds, monitored by analytical TLC. The obtained mixture was mixed with 200 mL of water and kept in the dark overnight, affording a dark oily phase and a clear aqueous layer, which was separated by filtration. Subsequently, the oil phase was dissolved in diethyl ether and extraction was carried out with NaOH (1%), obtaining an intense purple alkaline solution. HCl diluted was then added dropwise until purple color of the solution turned to orange, causing the formation of a precipitate. The solid formed was filtered, washed, extracted with hexane and purified by crystallization from ethanol/water.

*2-Hydroxy-3-[(1E)-prop-1-en-1-yl]naphthalene-1,4-dione* (**4a**). Orange scales; 41.5% yield; m.p. 135 °C; r.t.: 90 min; FTIR (KBr) ν: 3231.84, 2962.76, 1654.01, 1614.47, 1573 cm^−1^; ^1^H-NMR (400 MHz, CDCl_3_): δ 8.11 (dd, *J* = 24.9, 7.4 Hz, 2H), 7.73 (m, 3H), 7.09 (dd, *J* = 15.9, 7.0 Hz, 1H), 6.65 (d, *J* = 16.1 Hz, 1H), 2.01 (d, *J* = 6.6 Hz, 3H); ^13^C-NMR (600 MHz, CDCl_3_): δ 187.55 (C-1), 181.49 (C-4), 149.84 (C-2), 133.97 (C-12), 133.69 (C-7), 133.06 (C-6), 131.96 (C-10), 131.34 (C-9), 128.24 (C-8), 126.99 (C-5), 123.67 (C-11), 116.49 (C-3), 17.20 (C-13) ppm; MS *m*/*z* 214 [M^+^, C_13_H_10_O_3_].

*2-[(1E)-But-1-en-1-yl]-3-hydroxynaphthalene-1,4-dione* (**4b**). Orange powder; 47.5% yield; m.p. 107 °C; r.t.: 75 min; FTIR (KBr) ν: 3329.25, 3069.81, 3039.91, 2962.76, 2921.29, 2880.78, 1649.19, 1593.25 cm^−1^; ^1^H-NMR (400 MHz, CDCl_3_): δ 8.11 (dd, *J* = 24.8, 7.5 Hz, 2H), 7.74 (m, 3H), 7.13 (dt, *J* = 16.1, 6.7 Hz, 1H), 6.63 (d, *J* = 16.2 Hz, 1H), 2.24 (m, 2H), 1.67 (s, 14H), 1.14 (t, *J* = 7.4 Hz, 3H); ^13^C-NMR (600 MHz, CDCl_3_): δ 187.48 (C-1), 182.11 (C-4), 150.44 (C-3), 142.36 (C-12), 133.97 (C-7), 133.06 (C-6), 131.96 (C-9), 131.34 (C-10), 128.24 (C-8), 126.99 (C-5), 123.28 (C-11), 116.37 (C-2), 23.89 (C-13), 13.84 (C-14) ppm; MS: *m/z* 228 [M^+^, C_14_H_12_O_3_].

*2-[(1E)-Hex-1-en-1-yl]-3-hydroxynaphthalene-1,4-dione* (**4c**). Orange powder; 40.1% Yield; m.p. 85 °C; r.t.: 40 min; FTIR (KBr) ν: 3268.49, 2958.9, 2925.15, 2869.56, 1653.06, 1620.26, 1568.18 cm^−1^; ^1^H-NMR (400 MHz, CDCl_3_): δ 8.10 (dd, *J* = 25.1, 7.5 Hz, 2H), 7.77 (m, 3H), 7.09 (m, 1H), 6.63 (d, *J* = 16.2 Hz, 1H), 2.32 (q, *J* = 7.2 Hz, 2H), 1.44 (m, 4H), 0.93 (m, 3H); ^13^C-NMR (600 MHz, CDCl_3_): δ 187.48 (C-1), 182.11 (C-4), 150.44 (C-3), 140.39 (C-12), 133.97 (C-7), 133.06 (C-6), 131.96 (C-9), 131.34 (C-10), 128.24 (C-8), 127.81 (C-5), 126.99 (C-11), 116.37 (C-2), 31.63 (C-13), 31.33 (C-14), 21.86 (C-15), 14.02 (C-16) ppm; MS: *m*/*z* 256 [M^+^, C_16_H_16_O_3_].

#### 3.2.8. General Synthesis of Naphtho[2,3-*b*]furan-4,9-diones and Naphtho[1,2-*b*]furan-4,5-diones (**4d**–**4i**)

Two grams of **4a**, **4b** or **4c** were added in a solution of 6 g of mercuric acetate in 60 mL of acetic acid. The mixture was allowed to stir continuously for 30 min at r.t., subsequently warmed to 65 °C and allowed to cool to r.t., then the solvent was removed by rotaevaporation and ethyl ether was added to the concentrate. This solution was filtered to remove the non-soluble impurities and the ether was allowed to evaporate to form a very viscous, deep red liquid which was diluted in 30 mL of ethanol and 10 mL of concentrated HCl, then heated to 65 °C for 2 h with constant stirring. Once the reaction was completed and r.t. was reached, a pinch of activated carbon was added and filtered. Finally, 250 mL of water was added to the solution with caution, precipitating the compound (**4d**, **4e** or **4f**) as a yellow powder which was filtered, washed and dried. Compounds **4g**, **4h** and **4i** were obtained as an orange powder, following the same procedure described above, with changes in the amount of ethanol (40 mL instead of 30 mL), HCl (1 mL instead of 10 mL) and reaction time (15 min/65 °C instead of 2 h/65 °C).

*2-Methylnaphtho[2,3-b]furan-4,9-dione* (**4d**). Yellow powder; 21.3% yield; m.p. 174 °C; FTIR (KBr) ν: 2924.18, 1676.2, 1585.5, 1533.46, 1206.51 cm^−1^; ^1^H-NMR (400 MHz, CDCl_3_): δ 8.11 (m, 2H), 7.67 (m, 2H), 6.54 (t, *J* = 2.4 Hz, 1H), 2.44 (d, *J* = 0.7 Hz, 3H); ^13^C-NMR (101 MHz, CDCl_3_): δ 180.85 (C-4), 173.11 (C-9), 160.48 (C-2), 151.67 (C-13), 133.82 (C-6), 133.57 (C-7), 133.09 (C-12), 132.51 (C-10), 131.93 (C-11), 126.88 (C-8), 126.79 (C-5), 105.00 (C-3), 14.14 (C-14) ppm; MS: *m*/*z* 212 [M^+^, C_13_H_8_O_3_].

*2-Ethylnaphtho[2,3-b]furan-4,9-dione* (**4e**). Yellow powder; 42.3% yield; m.p. 143 °C; FTIR (KBr) ν: 3117.07, 2978.19, 2930.93, 1673.3, 1530.57, 1202.66 cm^−1^; ^1^H-NMR (400 MHz, CDCl_3_): δ 8.18 (m, 2H), 7.74 (p, *J* = 7.6 Hz, 2H), 6.63 (s, 1H), 2.86 (q, *J* = 7.5 Hz, 2H), 1.37 (t, *J* = 7.6 Hz, 3H); ^13^C-NMR (101 MHz, CDCl_3_): δ 180.91 (C-4), 173.17 (C-9), 165.88 (C-2), 151.56 (C-13), 133.78 (C-6), 133.53 (C-7), 133.09 (C-12), 132.57 (C-10), 131.78 (C-11), 126.85 (C-8), 126.78 (C-5), 103.51 (C-3), 21.80 (C-14), 11.60 (C-15) ppm; MS: *m*/*z* 226 [M^+^, C_14_H_10_O_3_].

*2-Butylnaphtho[2,3-b]furan-4,9-dione* (**4f**). Yellow powder; 39.2% yield; m.p. 104 °C; FTIR (KBr): ν: 3121.09, 3068.24, 2936.72, 2863.42, 1668.48, 1530.57, 1212.3 cm^−1^; ^1^H-NMR (400 MHz, CDCl_3_): δ 8.2 (m, 2H), 7.75 (m, 2H), 6.63 (s, 1H), 2.83 (t, *J* = 7.6 Hz, 2H), 1.76 (m, 2H), 1.43 (m, 2H), 0.98 (t, *J* = 7.4 Hz, 3H); ^13^C-NMR (101 MHz, CDCl_3_): δ 180.97 (C-4), 173.14 (C-9), 164.85 (C-2), 151.56 (C-13), 133.80 (C-6), 133.53 (C-7), 133.11 (C-12), 132.60 (C-10), 131.79 (C-11), 126.85 (C-8), 126.79 (C-5), 104.16 (C-3), 29.51 (C-14), 28.04 (C-15), 22.18 (C-16), 13.68 (C-17) ppm; MS: *m*/*z* 254 [M^+^, C_16_H_14_O_3_].

*2-Methylnaphtho[1,2-b]furan-4,5-dione* (**4g**). Orange powder; 12.3% Yield; m.p. 124 °C; FTIR (KBr) ν: 3122.86, 2923.22, 1673.3, 1554.68, 1215.19 cm^−1^; ^1^H-NMR (400 MHz, CDCl_3_): δ 8.04 (m, 1H), 7.63 (m, 2H), 7.43 (ddd, *J* = 7.7, 6.8, 2.0 Hz, 1H), 6.45 (d, *J* = 1.1 Hz, 1H), 2.43 (d, *J* = 1.1 Hz, 3H); ^13^C-NMR (101 MHz, CDCl_3_): δ 180.71 (C-5), 174.38 (C-4), 159.62 (C-2), 155.90 (C-13), 135.34 (C-8), 130.45 (C-10), 129.78 (C-6), 128.69 (C-7), 128.57 (C-11), 122.67 (C-9), 121.95 (C-12), 104.52 (C-3), 13.61 (C-14) ppm; MS: *m*/*z* 212 [M^+^, C_13_H_8_O_3_].

*2-Ethylnaphtho[1,2-b]furan-4,5-dione* (**4h**). Orange powder; 35.6% yield; m.p. 136 °C; FTIR (KBr): ν: 3119.96, 2976.26, 2927.26, 1668.48, 1549.86, 1208.44 cm^−1^; ^1^H-NMR (400 MHz, CDCl_3_): δ 8.03 (m, 1H), 7.63 (m, 2H), 7.42 (ddd, *J* = 7.7, 7.1, 1.7 Hz, 1H), 6.43 (m, 1H), 2.76 (qd, *J* = 7.5, 1.0 Hz, 2H), 1.32 (t, *J* = 7.5 Hz, 3H); ^13^C-NMR (101 MHz, CDCl_3_): δ 180.74 (C-5), 174.43 (C-4), 161.46 (C-2), 159.55 (C-13), 135.33 (C-8), 130.42 (C-10), 129.77 (C-6), 128.74 (C-7), 128.60 (C-11), 122.48 (C-9), 121.99 (C-12), 103.07 (C-3), 21.31 (C-14), 11.67 (C-15) ppm; MS: *m*/*z* 226 [M^+^, C_14_H_10_O_3_].

*2-Butylnaphtho[1,2-b]furan-4,5-dione* (**4i**). Orange powder; 32.7% Yield; m.p. 64 °C; FTIR (KBr): ν: 3109.36, 2943.47, 2861.49, 1668.48, 1552.75, 1206.51 cm^−1^; ^1^H-NMR (400 MHz, CDCl_3_): δ 8.03 (d, *J* = 7.5 Hz, 1H), 7.63 (qd, *J* = 7.6, 1.2 Hz, 2H), 7.42 (td, *J* = 7.7, 1.7 Hz, 1H), 6.44 (s, 1H), 2.72 (t, *J* = 7.5 Hz, 2H), 1.72 (tt, *J* = 15.3, 7.7 Hz, 2H), 1.44 (dq, *J* = 14.6, 7.4 Hz, 2H), 0.97 (m, 3H); ^13^C-NMR (101 MHz, CDCl_3_): δ 180.77 (C-5), 174.47 (C-4), 160.32 (C-2), 159.54 (C-13), 135.33 (C-8), 130.43 (C-10), 129.76 (C-6), 128.78 (C-7), 128.59 (C-11), 122.50 (C-9), 122.01 (C-12), 103.69 (C-3), 29.55 (C-14), 27.56 (C-15), 22.13 (C-16), 13.73 (C-17) ppm; MS: *m*/*z* 254 [M^+^, C_16_H_14_O_3_].

#### 3.2.9. Synthesis of Naphtho[2,3-*b*]furan-4,9-dione (**4j**)

Vinyl acetate and **1** in a molar relation 1:1, were dissolved in acetone. The mixture was irradiated with a mercury lamp of 450 W of high pressure at r.t. for 7 h, to yield a yellow precipitate. Subsequently, precipitate was reacted with p-TsOH in dry benzene at reflux temperature to afford yellow crystals; 65.6% yield; m.p. 229.4 °C; FTIR (KBr) ν: 3069, 1679, 1646, 1206 cm^−1^; ^1^H-NMR (600 MHz, CDCl_3_): δ 8.16 (m, 2H), 8.14 (d, 1H), 7.88 (m, 2H), 7.08 (d, 1H) ppm; ^13^C-NMR (600 MHz, CDCl_3_): δ 180.65 (C-4), 180.26 (C-9), 158.36 (C-13), 156.01 (C-2), 136.34 (C-6), 135.16 (C-7), 134.07 (C-12), 133.24 (C-10), 133.36 (C-11), 129.05 (C-8), 128.27 (C-5), 110.11 (C-3); MS *m*/*z* 198 [M^+^, C_12_H_6_O_3_].

#### 3.2.10. Synthesis of 2-Phenylnaphtho[2,3-*b*]furan-4,9-dione (**4k**)

Phenylacetylene (2.93 g) and **1** (500 mg) were dissolved in 115 mL of acetonitrile by stirring at r.t. This solution was then cooled to 0 °C and 3.16 g of CAN was added in portions to the yellow solution, which immediately turned orange, this solution was kept at 0 °C for 20 min until the reaction was quenched with saturated solution of NH_4_Cl. The reaction mixture was extracted with ethyl acetate (4 × 20 mL), washed with saturated solution of NaHCO_3_, and dried over anhydrous Na_2_SO_4_. The solvent was removed under reduced pressure and the crude product was purified by open column chromatography (SiO_2_; CH_2_Cl_2_) to afford yellow crystals; 18.2% yield, m.p. 220 °C; FTIR (KBr) ν: 3046, 1665, 1580, 1530, 1252 cm^−1^; ^1^H-NMR (600 MHz, CDCl_3_): δ 8.10 (m, 2H), 7.80 (m, 2H), 7.70 (m, 2H), 7.40 (m, 3H), 7.10 (s, 1H) ppm; ^13^C-NMR (600 MHz, CDCl_3_): δ 181.27 (C-4), 173.49 (C-9), 160.79 (C-2), 152.01 (C-13), 134.43 (C-6), 134.06 (C-7), 133.53 (C-10), 133.30 (C-11), 132.86 (C-12), 130.72 (C-1’), 129.54 (C-4’), 128.75 (C-3’; C-5’), 127.40 (C-8), 127.35 (C-5), 125.99 (C-2’; C-6’), 103.37 (C-3) ppm; MS *m*/*z* 274.27 [M^+^, C_18_H_10_O_3_].

#### 3.2.11. Synthesis of 2-Acetyl-2-methoxy-2,3-dihydronaphtho[2,3-*b*]furan-4,9-dione (**4l**)

To a solution of 1 g of **1** and 1.5 mL of 2,3-dimethoxy-1,3-butadiene in 60 mL of acetonitrile was added 9.58 g of CAN. The reaction was carried out at 0 °C for 6 h. The obtained mixture was diluted with water and extracted with ethyl acetate which was subsequently evaporated. The residue was purified by chromatography (SiO_2_; hexane/ethyl acetate, 5/1) to afford yellow powder; 51.43% yield; m.p. 145 °C; FTIR (KBr): ν: 3069, 2946, 2843, 1690, 1673, 1634, 1560, 1260 cm^−1^; ^1^H-NMR (600 MHz, CDCl_3_): δ 8.11 (m, 2H), 7.75 (m, 2H), 3.66 (d, 1H), 307 (d, 1H), 3.36 (s, 3H), 3.30 (s, 3H); ^13^C-NMR (600 MHz, CDCl_3_): δ 182.00 (C-14), 178.00 (C-4), 172.00 (C-9), 158.97 (C-13), 134.89 (C-6), 134.75 (C-7), 133.52 (C-10), 126.80 (C-11), 126.51 (C-8), 124.65 (C-5), 118.20 (C-12), 102.32 (C-2), 51.27 (C-16), 27.33 (C-3), 17.03 (C-15) ppm; MS *m*/*z* 272.25 [M^+^, C_15_H_12_O_5_].

#### 3.2.12. Synthesis of 2-Acetylnaphtho[2,3-*b*]furan-4,9-dione (**4m**)

To a solution of 266 mg of **4l** in 15 mL of benzene was added 0.3 mL of 1,8-diazabicyclo [5.4.0] undec-7-ene (DBU), the mixture was allowed to react at r.t. for 5 h. The reaction was quenched with aqueous ammonium chloride solution and extracted with ethyl acetate. After drying the solvent, the residue was purified by chromatography (SiO_2_; hexane/ethyl acetate, 4/1) initially, following by CH_2_Cl_2_ to afford yellow solid; 31% yield; m.p. 209 °C; FTIR (KBr): ν: 3113, 3017, 2940, 1690, 1672, 1582, 1262 cm^−1^; ^1^H-NMR (600 MHz, CDCl_3_): δ 8.20 (m, 2H), 7.77 (m, 2H), 7.53 (s, 1H), 2.59 (s, 1H) ppm; ^13^C-NMR (600 MHz, CDCl_3_): δ 188.23 (C-14), 180.29 (C-4), 174.44 (C-9), 155.76 (C-2), 153.33 (C-13), 134.96 (C-6), 134.83 (C-7), 133.45 (C-10), 132.94 (C-11), 131.10 (C-12), 127.76 (C-8), 127.62 (C-5), 112.95 (C-3), 27.04 (C-15) ppm; MS *m*/*z* 240.21 [M^+^, C_14_H_8_O_4_].

#### 3.2.13. Synthesis of Methyl 4,9-Dioxo-2-phenyl-2,3,4,9-tetrahydronaphtho[2,3-*b*]furan-3-carboxylate (**4n**)

To a solution of 200 mg of **1** in 46 mL of acetone was added 931 mg of methyl (2*E*) 2-phenylpropionate, and irradiated with pyrex^®^ glass filtered ultraviolet light at r.t. for 22 h, then the solvent was removed by evaporation under reduced pressure and the crude reaction product was purified by chromatography (SiO_2_; CH_2_Cl_2_) to afford yellow crystals; 20% yield; m.p. 167 °C; FTIR (KBr): ν: 3066, 3030, 2953, 1741, 1680, 1649, 1587, 1203 cm^−1^; ^1^H-NMR (600 MHz, CDCl_3_): δ 8.20 (m, 2H), 7.60 (m, 2H), 7.30 (s, 5H), 6.00 (dd, 1H), 4.30 (dd, 1H), 3.80 (s, 3H) ppm; ^13^C-NMR (600 MHz, CDCl_3_): δ 181.57 (C-4), 178.01 (C-9), 171.33 (C-14), 160.86 (C-13), 138.55 (C-1’), 134.95 (C-6), 133.71 (C-7), 133.36 (C-10), 132.02 (C-11), 129.72 (C-8), 129.51 (C-4’), 126.99 (C-3’; C-5’), 126.80 (C-2’; C-6’), 125.98 (C-5), 122.06 (C-12), 90.06 (C-2), 54.57 (C-3), 53.67 (C-15) ppm; MS *m*/*z* 334.32 [M^+^, C_20_H_14_O_5_].

#### 3.2.14. General Synthesis of Dihydronaphtho[2,3-*b*]furan-4,9-diones and Dihydronaphtho[1,2-b]furan-4,5-diones (**4o**–**4p**)

To a solution of 174 mg of **1** and 0.6 mL of 2,3-dimethyl-1,3-butadiene in 25 mL of acetonitrile, 1.206 g of CAN and sodium bicarbonate were added at 0 °C, allowing to react for 3 h. The obtained mixture was extracted with ethyl acetate (3 × 100 mL), the solvent was dried and the residue purified by chromatography (SiO_2_; CH_2_Cl_2_).

*2-Methyl-2-(prop-1-en-2-yl)-2,3-dihydronaphtho[2,3-b]furan-4,9-dione* (**4o**). Yellow powder; 47.63% yield; m.p. 135 °C; FTIR (KBr): ν: 3097, 3070, 2985, 2929, 2860, 1680, 1643, 1590, 1254 cm^−1^; ^1^H-NMR (600 MHz, CDCl_3_): δ 8.0 (m, 2H), 7.62 (m, 2H), 5.05 (d, 1H), 4.86 (d, 1H), 3.14 (d, 1H), 2,96 (d, 1H), 1.78 (s, 3H), 1.59 (s, 3H) ppm; ^13^C-NMR (600 MHz, CDCl_3_): δ 182.91 (C-4), 178.49 (C-9), 159.38 (C-13), 145.99 (C-14), 134.54 (C-6), 133.35 (C-7), 132.09 (C-10), 126.76 (C-11), 126.75 (C-8), 123.81 (C-5), 117.72 (C-12), 111.73 (C-15), 95.01 (C-2), 39.14 (C-3), 26.67 (C-17), 18.86 (C-16) ppm; MS *m*/*z* 254.28 [M^+^, C_16_H_14_O_3_].

*2-Methyl-2-(prop-1-en-2-yl)-2,3-dihydronaphtho[1,2-b]furan-4,5-dione* (**4p**). Red powder; 40.55% yield; m.p. 147 °C; FTIR (KBr): ν: 3066, 2980, 2932, 1700, 1647, 1625, 1590, 1254 cm^−1^; ^1^H-NMR (600 MHz, CDCl_3_): δ 8.03 (d, 1H), 7.6 (m, 3H), 5.11 (s, 1H), 4.95 (s, 1H), 3.12 (d, 1H), 2.86 (d, 1H), 1.78 (s, 3H), 1.61 (s, 3H) ppm; ^13^C-NMR (600 MHz, CDCl_3_): δ 181.59 (C-4), 176.03 (C-5), 169.03 (C-13), 146.17 (C-14), 134.96 (C-8), 132.38 (C-10), 131.34 (C-6), 129.94 (C-7), 129.91 (C-9), 128.18 (C-11), 115.35 (C-12), 111.55 (C-15), 96.63 (C-2), 38.36 (C-3), 26.77 (C-17), 18.91 (C-16) ppm; MS *m*/*z* 254.3 [M^+^, C_16_H_14_O_3_].

#### 3.2.15. General Synthesis of Dihydronaphtho[2,3-*b*]furan-4,9-diones and Dihydronaphtho[1,2-*b*]furan-4,5-diones (**4q**–**4r**)

Compound **1** (500 mg) and styrene (3 g) were dissolved in 115 mL of acetonitrile. This solution was then cooled to 0 °C and 3.16 g (5.74 mmol) of CAN was added in portions to the yellow solution, which immediately turned orange, this solution was kept at 0 °C for 20 m until the reaction was quenched with saturated solution of NH_4_Cl. After evaporation of the solvent, the crude product was extracted with ethyl acetate. The organic phase was washed successively with saturated sodium bicarbonate solution and saline solution and dried over anhydrous Na_2_SO_4_. After removal of the solvent under reduced pressure, the crude product was purified by chromatography (SiO_2_; CH_2_Cl_2_) initially, followed by (SiO_2_; hexane/ethyl acetate, 4/1).

*2-Phenyl-2,3-dihydronaphtho[2,3-b]furan-4,9-dione* (**4q**). Yellow crystals; 27% yield; m.p. 158 °C; FTIR (KBr): ν: 3057, 3010, 1674, 1647, 1594, 1243 cm^−1^; ^1^H-NMR (600 MHz, CDCl_3_): δ 8.00 (m, 2H), 7.60 (m, 2H), 7.30 (s, 5H), 6.00 (dd, 1H), 3.60 (dd, 1H), 3.20 (dd, 1H) ppm; ^13^C-NMR (600 MHz, CDCl_3_): δ 182.65 (C-4), 178.17 (C-9), 160.28 (C-13), 139.96 (C-1’), 134.66 (C-6), 133.51 (C-7), 132.04 (C-10), 129.35 (C-11), 126.83 (C-8), 126.55 (C-4’), 126.43 (C-3’; C-5’), 124.29 (C-2’; C-6’), 123.64 (C-5), 123.57 (C-12), 87.22 (C-2), 35.72 (C-3) ppm; MS *m*/*z* 276.28 [M^+^, C_18_H_12_O_3_].

*2-Phenyl-2,3-dihydronaphtho[1,2-b]furan-4,5-dione* (**4r**). Orange crystals; 26% yield; m.p. 115 °C; FTIR (KBr): ν: 3062, 3035, 1652, 1618, 1588, 1240 cm^−1^; ^1^H-NMR (600 MHz, CDCl_3_): δ 8.10 (m, 1H), 7.60 (m, 3H), 7.30 (s, 5H), 6.00 (dd, 1H), 3.50 (dd, 1H), 3.10 (dd, 1H) ppm; ^13^C-NMR (600 MHz, CDCl_3_): δ 182.00 (C-4), 175.86 (C-5), 169.93 (C-13), 139.93 (C-1’), 135.03 (C-8), 132.53 (C-10), 131.24 (C-6), 130.04 (C-7), 129.55 (C-4’), 129.49 (C-3’; C-5’), 127.88 (C-2’; C-6’), 126.48 (C-11), 125.11 (C-9), 115.55 (C-12), 88.79 (C-2), 34.93 (C-3) ppm; MS *m*/*z* 276.28 [M^+^, C_18_H_12_O_3_].

#### 3.2.16. Synthesis 2,2,3,3-Tetramethyl-2,3-dihydronaphtho[1,2-*b*]furan-4,5-dione (**4s**)

To a solution of 500 mg of **1** and 2.42 g of 2,3-dimethyl-2-butene in 115 mL of acetonitrile was cooled to 0 °C and 3.16 g of CAN were added in portions to the yellow solution which immediately turned orange, this solution was kept at 0 °C for 20 min until the reaction was quenched with saturated solution of NH_4_Cl. After evaporation of the solvent under reduced pressure, the crude product was extracted with ethyl acetate (3 × 100 mL). The organic phase was washed successively with saturated solution of NaHCO_3_, then with saline and dried over anhydrous Na_2_SO_4_. After removal of the solvent under reduced pressure, the crude product was separated by chromatography (SiO_2_; CH_2_Cl_2_) to afford orange crystals; 2.5% yield; m.p. 91 °C; FTIR (KBr): ν: 3090, 2980, 1963, 1695, 1638, 1614, 1588 cm^−1^; ^1^H-NMR (600 MHz, CDCl_3_): δ 8.06 (m, 1H), 7.65 (m, 2H), 7.58 (m, 1H), 1.48 (s, 6H), 1,36 (s, 6H) ppm; ^13^C-NMR (600 MHz, CDCl_3_): δ 181.73 (C-4), 175.63 (C-5), 167.40 (C-13), 134.36 (C-8), 131.58 (C-10), 130.91 (C-6), 129.13 (C-7), 128.26 (C-11), 124.44 (C-9), 123.23 (C-12), 97.23 (C-2), 46.35 (C-3), 23.17 (C-14; C-15), 22.01 (C-16; C-17) ppm; MS *m*/*z* 256.30 [M^+^, C_16_H_16_O_3_].

#### 3.2.17. Synthesis of 2-Ethenyl-2-methyl-2,3-dihydronaphtho[1,2-*b*]furan-4,5-dione (**4t**)

To a solution of 174 mg of **1** and 0.6 mL of isoprene in 25 mL of acetonitrile was added 1.206 g of CAN and sodium bicarbonate at 0 °C, allowing reacting for 3 h. The obtained mixture was extracted with ethyl acetate, the solvent was dried and the residue purified by flash chromatography (SiO_2_; CH_2_Cl_2_) to afford yellow powder; 48.75% yield; m.p. 150 °C; FTIR (KBr): ν: 3068, 2926, 1693, 1643, 1612, 1586, 1251 cm^−1^; ^1^H-NMR (600 MHz, CDCl_3_): δ 8.09 (d, 1H), 7.69 (m, 3H), 6.10 (dd, 1H), 5.39 (d, 1H), 5.26 (d, 1H), 3.17 (d, 1H), 3.02 (d, 1H), 1.70 (s, 3H), ppm; ^13^C-NMR (600 MHz, CDCl_3_): δ 181.11 (C-4), 175.53 (C-5), 168.44 (C-13), 139.79 (C-14), 134.51 (C-8), 132.05 (C-10), 130.09 (C-6), 129.60 (C-7), 127.75 (C-11), 124.68 (C-9), 114.19 (C-15), 93.86 (C-12), 89.96 (C-2), 38.34 (C-3), 26.95 (C-16) ppm; MS *m/z* 240.25 [M^+^, C_15_H_12_O_3_].

#### 3.2.18. Synthesis of 4,9-Dimethoxy-4,9-dihydronaphtho[2,3-*d*][1,3]thiazole (**5a**)

To a solution of 4.30 g (20 mmol) of **5** and 65 mg (0.20 mmol) tetrabutylammonium bromide in 150 mL of dry THF under N_2_, 34.82 g (200 mmol) sodium dithionite in 100 mL of water was added dropwise. The mixture was vigorously stirred for 30 min and then treated with 22.44 g (400 mmol) KOH in 70 mL of water. After 30 min, it was cooled to 0 °C on an ice-bath, 22.8 mL of dimethyl sulfate (30.27 g, 240 mmol) was added, and the mixture was then stirred for 2 h at 0 °C. Then, it was allowed to warm at rt and stirred for 2 h. Ammonia solution (10%, 50 mL) was added, and the mixture was stirred for 30 min and then extracted with CH_2_Cl_2_ (3 × 100 mL). The combined organic phase was washed with water (150 mL), then with a saturated solution of NaCl (150 mL), dried over Na_2_SO_4_ and evaporated. The residue was purified by chromatography (SiO_2_; CH_2_Cl_2_) to afford beige crystals; 77% yield; m.p. 85 °C; FTIR (KBr): ν: 2935, 1582 cm^−1^; ^1^H-NMR (400 MHz, CDCl_3_): δ 8.93 (s, 1H,), 8.43 (m, 1H), 8.21 (m, 1H), 7.51 (m, 2H), 4.42 (s, 3H), 4.11 (s, 3H); ^13^C-NMR (400 MHz, CDCl_3_): δ 153.36 (C-2), 146.14 (C-12), 145.33 (C-4), 142.78 (C-9), 126.51 (C-13), 126.31 (C-10), 125.86 (C-11), 125.49 (C-6), 124.09 (C-7), 123.43 (C-8), 121.17 (C-5), 63.07 (C-14), 60.81 (C-15) ppm; MS *m*/*z* 245.29 [M^+^, C_13_H_11_O_2_NS].

#### 3.2.19. Synthesis of Methyl 4,9-dimethoxy-4,9-dihydronaphtho[2,3-*d*][1,3]thiazole-2-carboxylate (**5b**)

Following a procedure reported by Vechorkin et al. [18], 2.50 g (10.20 mmol) **5a** and 4.00 g (12.28 mmol) Cs_2_CO_3_ were evacuated in a dried Schlenck flask for 1 h. Then, anhydrous DMF (20 mL) was added under N_2_, and the reaction vessel was degassed and flushed alternately with N_2_ and CO_2_ three times each. Then, the reaction mixture was heated at 125 °C under an overpressure of CO_2_ (400 mbar) for 16 h. Thereafter, the reaction mixture was allowed to reach 60 °C, methyl iodide (1.87 mL, 4.26 g, 30 mmol) was added with a syringe through a septum, and the mixture was stirred for an additional 2 h at 60 °C. Then, it was cooled to r.t., water (20 mL) was added, and the mixture was extracted with CH_2_Cl_2_ (3 × 30 mL). The combined organic phase was washed with a saturated solution of NaCl (40 mL) and dried over Na_2_SO_4_. The solvent was evaporated and the residue purified by chromatography (SiO_2_; CH_2_Cl_2_/hexane, 9/1) to afford orange crystals; 64% yield; m.p. 150 °C; FTIR (KBr): ν: 2970, 1713, 1574 cm^−1^; ^1^H-NMR (600 MHz, CDCl_3_): δ 8.46 (d, ^3^*J* = 8.7 Hz, 1H), 8.20 (d, ^3^*J* = 8.6 Hz, 1H), 7.60 (dd, ^3^*J*_1_ = 8.7 Hz, ^3^*J*_2_ = 6.7 Hz, 1H), 7.54 (dd, ^3^*J*_1_ = 8.6 Hz, ^3^*J*_2_ = 6.7 Hz, 1H), 4.51 (s, 3H), 4.11 (s, 3H), 4,09 (s, 3H); ^13^C-NMR (600 MHz, CDCl_3_): δ 161.35 (C-14), 156.64 (C-2), 148.40(C-9), 144.71 (C-12), 142.02 (C-4), 127.42 (C-13), 127.18 (C-10), 126.55 (C-11), 126.22 (C-6), 125.75 (C-7), 124.00 (C-8), 121.24 (C-5), 63.51 (C-16), 60.93 (C-17), 53.99 (C-15) ppm; MS *m*/*z* 318.36 [M^+^, C_16_H_16_O_4_NS].

#### 3.2.20. Synthesis of Methyl 4,9-Dioxo-4,9-dihydronaphtho[2,3-*d*][1,3]thiazole-2-carboxylate (**5c**)

To a suspension of **5b** (0.10 g, 0.33 mmol) in MeCN (1 mL) and water (4 mL) at 0 °C was added dropwise within 20 min (NH_4_)_2_[Ce(NO_3_)_6_] (0.51 g, 0.92 mmol) in MeCN (4 mL) and water (4 mL) under vigorous stirring. The mixture was allowed to react for 30 min under the same conditions. Then it was poured onto ice-water (20 mL) and extracted with CH_2_Cl_2_ (3 × 30 mL). The combined organic phase was washed with a saturated solution of NaCl (2 × 50 mL), dried over Na_2_SO_4_, then concentrated, and purified by chromatography (SiO_2_; CH_2_Cl_2_) to afford a yellow solid; 43% yield; m.p. 175–176 °C; FTIR (KBr): ν: 2966, 1720, 1678, 1585 cm^−1^; ^1^H-NMR (CDCl_3_, 400 MHz) δ 8.34 (dd, ^3^*J* = 7.5 Hz, ^4^*J* = 1.6 Hz, 1H), 8.24 (dd, ^3^*J* = 7.3 Hz, ^4^*J* = 1.8 Hz, 1H), 7.83 (m, 2H), 4.08 (s, 3H,); ^13^C-NMR (CDCl_3_, 600 MHz) δ 178.50 (C-9), 177.22 (C-4), 164.25 (C-14), 159.96 (C-2), 155.12 (C-12), 145.76 (C-10), 135.33 (C-11), 134.69 (C-7), 133.26 (C-6), 133.20 (C-13), 128.46 (C-8), 127.67 (C-5), 54.38 (C-15) ppm; MS *m*/*z* 288.3 [M^+^, C_14_H_10_O_4_NS].

### 3.3. Biological Evaluation

#### 3.3.1. Cell Culture

The synthesized compounds were evaluated for their in vitro cytotoxic activities against a human colorectal cancer cell line (HT-29) and normal dermal fibroblasts (PCS-201-012), obtained from the American Type Culture Collection (ATCC; Manassas, VA, USA). Cells were maintained routinely in McCoy’s 5A (HT-29) or Dulbecco’s Modified Eagle's medium (PCS-201-012), supplemented with 10% fetal bovine serum, sodium bicarbonate (1.5 g/L), penicillin (100 UI/mL), streptomycin (100 µg/mL), and low serum growth supplement (Invitrogen) for dermal fibroblasts, at 37 °C and 5% CO_2_.

#### 3.3.2. Preparation of Test Compounds

Test compounds were dissolved in DMSO to obtain stock solutions that were maintained as small aliquots at −20 °C. At time of drug addition, an aliquot was thawed and diluted to obtain the desired final maximum test concentration of 100 μM, with complete medium. The final percentage of DMSO in the wells was less than 1% (*v*/*v*). The racemic mixtures of compounds **3b**, **4l**, **4n**, **4o**, **4p**, **4q**, **4r** and **4t** were evaluated because their isomers were not available separately.3.3.3. Anti-Proliferative Assay

The effect of tests compounds on cell viability was evaluated using the 3-(4,5-dimethylthiazol-2-yl)-2,5-diphenyltetrazolium bromide (MTT) reduction assay [27], Briefly, HT-29 cells were cultured at 37 °C and 5% CO_2_, with various concentrations of quinone derivatives (0–100 µM). Doxorubicin, a drug widely used to treat different types of cancer, was used as a positive control. After 48 h, the medium was removed and cells were incubated with MTT solution (0.25 mg/mL). Four hours later, the medium was carefully aspired and formazan crystals were dissolved in DMSO (100 μL). The OD_550_ was measured using a plate reader (Multiskan Go, Thermo Scientific, Waltham, MA, USA). Inhibition of cell proliferation was expressed as percentage of viable cells in treated samples as compared to vehicle-treated cells and the 50% inhibitory concentration of cell proliferation (IC_50_) was calculated by nonlinear regression. The selectivity index (SI), was calculated as a ratio of IC_50Fibroblast_/IC_50HT-29cells_.

### 3.4. 3D-QSAR Study

**Figure 6:**
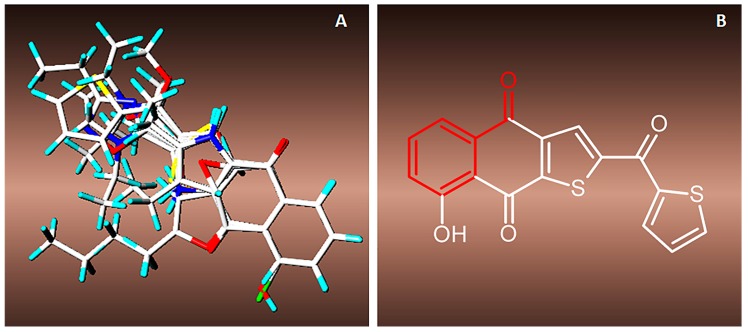
(**A**) Alignment of the training and test sets of quinone derivatives using compound **7** as a template; and (**B**) the pharmacophore was defined as a common substructure for all studied molecules and highlighted in the structure of compound **7**.

A three-dimensional quantitative structure–activity relationship study (3D-QSAR) was carried out, using comparative molecular field analysis (CoMFA), with 18 molecules (**1a**, **2**, **2c**, **2d**, **3c**, **4d**, **4e**, **4f**, **4g**, **4h**, **4i**, **4j**, **4s**, **5c**, **6**, **7**, **8** and **9**) with defined stereochemistry, which showed cytotoxic activity on HT-29 cell line (IC_50_ < 100 μM). Molecules were digitized and optimized (Hartree-Fock method with base function 3-21G) using the GaussView 5.0 program (Gaussian Inc., Wallingford, CT, USA) [28]. The resulting geometry was converted to Mol2 format using Open Babel 2.3.1 software [29]. Geometry of compound **7**, the most active molecule of the series, was used as a reference for the alignment, and the ring highlighted in red on this compound was taken as a common substructure (Figure 6). The QSAR Project Manager module of SYBYL-X 2.0 program package (Tripos, St. Louis, MO, USA), was used to generate the CoMFA model, in which cytotoxic activity (−logCL_50_ (pIC_50_)), of the set of molecules were correlated with its structural parameters. The partial least squares method (PLS) was used to correlate the structural changes (CoMFA fields) with the changes in the biological activity of the quinone derivatives. The cross-validation of the model was carried out using the Leave-One-Out method (LOO) [30].

### 3.5. Statistical Analysis

The results are expressed as the mean of triplicate measurements in at least two independent experiments. Concentrations that inhibited cell survival (IC_50_) were calculated employing nonlinear regression and are shown together with their 95% confidence interval (IC).

## 4. Conclusions

In this work, we synthesized a series of naphthoquinone derivatives and evaluated their anti-proliferative activity against the human cell line of colorectal cancer HT-29. Several of these compounds exhibited significant anti-proliferative activity, with compound 7 being the most active of the series. A SAR analysis suggests that the activity is improved in molecules with tricyclic naphtho[2,3-*b*]furan-4,9-dione and naphtho[2,3-*b*]thiophene-4,9-dione systems 2-substituted with an electron-withdrawing group. On the other hand, the CoMFA analysis of steric and electrostatic fields showed that bulky groups as the thiophenyl group (ring D), at the C-2 of the naphtho[2,3-*b*]thiophene-4,9-dione system, have a positive influence on the anti-proliferative activity of these compounds, which is enhanced when ethyl groups are included in C-3 and C-4 of the ring D. Taking into account these results, we proposed five new compounds with better theoretical activity than the parental compound (**7**). From the set of designed compounds, **7d** presented the best theoretical pharmacological potential, which warrants its synthesis and biological evaluation. Further investigations are needed to determine the mechanism involved in the cytotoxic effect of the most active compounds which are potential candidates for new anti-CRC agents.

## Figures and Tables

**Table 1:** Cytotoxic activity of quinone derivatives against HT-29 colorectal cancer cells and fibroblasts PCS-201-012.

Compound	HT-29 IC_50_ μM (95% CI ^1^)	PCS-201-012 IC_50_ μM (95% CI)	Selectivity Index ^2^
**1a**	10.76 (9.72–11.86)	8.17 (7.35–9.21)	0.76
**2**	24.60 (20.89–28.33)	7.34 (6.35–8.44)	0.30
**2a**	>100	---	nd^3^
**2b**	>100	---	nd
**2c**	40.18 (32.43–48.74)	79.39 (61.54–102.41)	1.98
**2d**	68.71 (55.88–83.93)	51.53 (35.90–73.74)	0.75
**2e**	>100	---	nd
**2f**	>100	---	nd
**3a**	>100	---	nd
**3b** ^4^	57.29 (46.39–70.40)	28.42 (22.24–36.32)	0.50
**3c**	11.02 (10.29–11.84)	9.92 (9.19–10.74)	0.90
**4a**	>100	---	nd
**4b**	>100	---	nd	
**4c**	>100	---	nd
**4d**	17.62 (14.83–21.30)	18.49 (14.58–23.52)	1.05
**4e**	24.95 (21.01–29.79)	20.09 (16.03–25.03)	0.80
**4f**	21.68 (17.68–26.17)	10.00 (7.79–12.92)	0.46
**4g**	8.36 (7.10–9.80)	1.93 (1.66–2.24)	0.23
**4h**	13.68 (12.36–15.07)	3.68 (3.16–4.29)	0.27
**4i**	9.16 (7.99–10.45)	3.86 (3.16–4.70)	0.42
**4j**	8.20 (7.22–9.30)	8.28 (7.16–9.52)	1.01
**4k** ^5^	>10	---	nd
**4l** ^4^	34.85 (30.06–40.54)	12.87 (9.29–17.63)	0.37
**4m**	4.61 (3.72–5.67)	3.50 (2.92–4.21)	0.76
**4n** ^4^	>100	---	nd
**4o** ^4^	25.79 (21.08–31.83)	47.88 (38.63–58.55)	1.86
**4p** ^4^	28.08 (24.26–32.39)	4.19 (3.63–4.86)	0.15
**4q** ^4^	30.25 (25.4136.13)	9.16 (7.90–10.70)	0.30
**4r** ^4^	14.76 (12.10–18.04)	8.20 (6.53–10.29)	0.56
**4s**	18.11 (16.48–20.02)	19.94 (15.44–26.35)	1.10
**4t** ^4^	88.10 (79.74–96.52)	19.69 (17.39–22.28)	0.22
**5c**	13.90 (11.60–16.69)	7.26 (6.33–8.30)	0.52
**6**	2.67 (2.26–3.18)	1.49 (1.26–1.75)	0.56
**7**	1.73 (1.48–2.02)	4.56 (3.63–5.75)	2.63
**8**	6.89 (5.66–8.29)	2.56 (2.23–2.98)	0.37
**9**	3.07 (2.54–3.69)	1.03 (0.81–1.33)	0.34
Doxorubicin	1.07 (0.86–1.30)	---	nd

^1^ Confidence interval. ^2^ Selectivity index (SI) = IC_50Fibroblast_/IC_50HT-29cells_. ^3^ nd. Not determined. ^4^ Racemic mixtures. ^5^ Compound **4k** could not be evaluated at concentrations higher to 10 μM due to lack of solubility with DMSO.

**Table 2:** Experimental and predicted values of pIC_50_ obtained with the best comparative molecular field analysis (CoMFA) model for the training and test sets.

Compound	pIC_50_ Experimental	pIC_50_ Predicted
**1a**	4.968	4.968
**2**	4.609	4.592
**2c**	4.396	4.429
**2d**	4.163	4.147
**4d**	4.754	4.782
**4e**	4.603	4.617
**4g**	5.078	5.020
**4h**	4.864	4.952
**4i**	5.038	5.039
**4j**	5.086	5.035
**5c**	4.857	4.808
**6**	5.574	5.567
**7**	5.762	5.720
**8**	5.162	5.166
**9**	5.513	5.586
**3c** ^1^	4.958	4.897
**4f** ^1^	4.664	4.973
**4s** ^1^	4.742	4.974

^1^ Compounds in the test set.

**Table 3:** Predicted cytotoxic activity for the newly designed structures using the comparative molecular field analysis (CoMFA) model.

Compound	pIC_50_ Predicted	IC_50_ Predicted (μM)
**7a**	6.2054	0.623
**7b**	6.1853	0.653
**7c**	6.3186	0.480
**7d**	6.3884	0.409
**7e**	6.3361	0.461
